# PTML Modeling for Pancreatic Cancer Research: In Silico Design of Simultaneous Multi-Protein and Multi-Cell Inhibitors

**DOI:** 10.3390/biomedicines10020491

**Published:** 2022-02-18

**Authors:** Valeria V. Kleandrova, Alejandro Speck-Planche

**Affiliations:** 1 Laboratory of Fundamental and Applied Research of Quality and Technology of Food Production, Moscow State University of Food Production, Volokolamskoe Shosse 11, 125080 Moscow, Russia; valeria.kleandrova@gmail.com; 2 Grupo de Química Computacional y Teórica (QCT-USFQ), Departamento de Ingeniería Química, Universidad San Francisco de Quito, Diego de Robles y vía Interoceánica, Quito 170901, Ecuador

**Keywords:** caspase-1, cell line, fragment, IGF1R, MLP, multi-target, pancreatic cancer, TNF-alpha, virtual design

## Abstract

Pancreatic cancer (PANC) is a dangerous type of cancer that is a major cause of mortality worldwide and exhibits a remarkably poor prognosis. To date, discovering anti-PANC agents remains a very complex and expensive process. Computational approaches can accelerate the search for anti-PANC agents. We report for the first time two models that combined perturbation theory with machine learning via a multilayer perceptron network (PTML-MLP) to perform the virtual design and prediction of molecules that can simultaneously inhibit multiple PANC cell lines and PANC-related proteins, such as caspase-1, tumor necrosis factor-alpha (TNF-alpha), and the insulin-like growth factor 1 receptor (IGF1R). Both PTML-MLP models exhibited accuracies higher than 78%. Using the interpretation from one of the PTML-MLP models as a guideline, we extracted different molecular fragments desirable for the inhibition of the PANC cell lines and the aforementioned PANC-related proteins and then assembled some of those fragments to form three new molecules. The two PTML-MLP models predicted the designed molecules as potentially versatile anti-PANC agents through inhibition of the three PANC-related proteins and multiple PANC cell lines. Conclusions: This work opens new horizons for the application of the PTML modeling methodology to anticancer research.

## 1. Introduction

Pancreatic cancer (PANC) is currently recognized as the seventh most significant cause of cancer-related deaths worldwide. In addition to being associated with a very poor prognosis, PANC presents five highly alarming aspects. First, the mortality rate of PANC almost equals its incidence rate, since PANC accounted for 458,918 new cases and 432,242 deaths in 2018 [1]. This is consistent with the overall 5-year survival rate of approximately 6%, which makes PANC the most lethal cancer of all [2]. Second, the global burden involving this intractable neoplasm has more than doubled over the past 25 years [3]. Third, PANC is characterized by the emergence of drug resistance [4]; this makes PANC difficult to treat. Four, from a genetic point of view, PANC is very complex because PANC predisposition genes are not well understood, although several genes suggested to be involved in PANC development and progression have been studied [5]. Last, the current chemotherapeutic drugs used to treat PANC are limited in terms of effectiveness because they are mainly available as adjuvants and act through specific mechanisms of action. All these aspects indicate the urgent need for new and more effective anti-PANC chemotherapeutics able to act as multi-target drugs, simultaneously inhibiting several PANC-related proteins.

To date, some of the biomolecular targets studied in PANC research are proteins such as caspase-1, tumor necrosis factor-alpha (TNF-alpha), and insulin-like growth factor-1 receptor (IGF1R), which are promising biomolecular targets against PANC. In the case of caspase-1 and TNF-alpha, they are key proteins in inflammatory processes, with the former initiating inflammatory responses through the release of diverse proinflammatory cytokines [6,7,8] and pyroptosis (a form of programmed lytic cell-death) [9,10] and the latter being a proinflammatory cytokine capable to triggering other inflammation-related proteins (caspase-1 included) [11]. Notice that inflammation has long been accepted as a key component of carcinogenesis because, during inflammation, inflammasomes are potent contributors to cancer progression [12]. In the context of PANC, it has been demonstrated that the inhibition or deletion of components of the NLRP3 inflammasome, such as caspase-1, decreases tumor growth and metastasis in PANC by reprogramming innate and adaptive immunity in the tumor microenvironment [13]. It has also been proven that the inhibition of caspase-1 induced cell death in PANC cells [14]. Additionally, in the case of TNF-alpha, its inhibition led to the diminution of desmoplasia and inflammation to overcome chemoresistance in PANC [15]. In the case of IGF1R, high expression levels of this protein are associated with high tumor grade and poor survival [16], while targeting it inhibited PANC growth and metastasis [17]. In clinical trials, the combination of the well-known anticancer drug gemcitabine with an IGF1R inhibitor (Ganitumab) resulted in a numerical improvement compared to gemcitabine plus placebo [18].

The experimental evidence explained above suggests that the simultaneous inhibition of caspase-1, TNF-alpha, and IGF1R by a multi-target agent could constitute a promising alternative against future PANC treatment. In this sense, finding such multi-target agents can be accelerated utilizing the methodology known as perturbation theory and machine learning (PTML), which allows the integration of different kinds of chemical and biological data [19,20,21,22,23] and has been successfully applied to different drug discovery areas, such as oncology [24,25,26], neuroscience [27,28,29,30], immunology and immunotoxicity [31,32], infectious diseases [33,34,35,36,37,38], and drug delivery [39]. Considering all the ideas mentioned until now, in this work we establish the theoretical foundations for the rational discovery of multi-target chemicals against caspase-1, TNF-alpha, and IGF1R. Particularly, we report, for the first time, two PTML models based on multi-layer perceptron networks (PTML-MLP) to perform virtual design and prediction of molecules that can simultaneously inhibit not only the aforementioned proteins but also multiple PANC cell lines.

## 2. Materials and Methods

### 2.1. Bioactivity Data and Molecular Descriptors

All the steps necessary for the creation of a PTML-MLP model have been described in detail very recently [37,40]. Therefore, we will focus on the specific aspects of the two PTML-MLP models reported here. Chemical and biological data based on protein inhibition were retrieved from the ChEMBL database [41], while growth inhibition data on PANC cell lines were extracted from the public repository known as Genomics of Drug Sensitivity in Cancer (GDSC) v7.0 [42]. Our dataset contained 3833 different chemicals, each of them experimentally tested by considering at least 1 out of 2 measures of inhibitory activity (*ma*), defined as IC_50_ (nM)p (the concentration required for 50% inhibition of a protein) and IC_50_ (nM)c (the concentration required for a chemical to inhibit cell viability by 50%). Simultaneously, in these experimental assays, each chemical was tested against at least 1 out of 34 biomolecular or cellular targets (*tg*) while involving at least 1 out of 5 types of assay information (*ei*). Notice that each combination of the elements *ma*, *tg*, and *ei*, defined a unique experimental condition, *cj* (denoted as *cj*(*ma*, *tg*, and *ei*)), under which a chemical was assayed. Most of the chemicals were tested in one *cj*. Therefore, after removing entries containing duplicates (only keeping the ones with the lowest inhibition values among the duplicates), as well as those lacking SMILES, activity values, or measurement units, our dataset remained with 9705 statistical cases.

We selected certain cutoff values (Table 1) of inhibitory activity to annotate each case/chemical of our dataset as active (*IAi*(*cj*) = 1) or inactive (*IAi*(*cj*) = –1), with *IAi*(*cj*) being a dichotomous variable that indicated the activity of the *i*th case/chemical under a defined *cj*. These cutoff values were rigorous enough (lower than the 10 µM used in high-throughput screening campaigns) [43] and prevented an unnecessary imbalance between the numbers of cases/chemicals annotated as active and those considered inactive.

For the case of the first PTML-MLP model (*Model 1*), we used the SMILES codes of the 9705 cases/chemicals (already stored in a txt file) and calculated the topological descriptors known as spectral moments of the bond adjacency matrix (*SM*(*PP*)k) [44,45,46], atom-based connectivity indices *X*(*s*)o [47,48], atom-based valence connectivity indices *Xv*(*s*)o [48,49], and bond connectivity indices *e*(*s*)o [50]. In this sense, for the topological descriptor *SM*(*PP*)k, the term “k” is the *k*th power of the bond adjacency matrix and “*PP*” is an atomic physicochemical property, such as hydrophobicity (*Hyd*), polar surface area (*Psa*), molar refractivity (*Mol*), Gasteiger–Marsili charges (*Gas*), and atomic weight (*Ato*). In *X*(*s*)o, *Xv*(*s*)o, and *e*(*s*)o, the term “*s*” represents the type of subgraph/fragments, such as paths (P), clusters (C), path-clusters (PC), and chains (Ch), while the term “o” is the order of the topological descriptor and indicates the number of bonds (without counting bond multiplicity) of each subgraph/fragment. All these topological descriptors were calculated by the software MODESLAB v1.5 [51,52]. Moreover, size-independent topological descriptors (*NTI*) were calculated according to the following formalism:

(1) $$NTI=\frac{TI}{nB}\;\;\;\;\;\;$$

In Equation (1), *TI* represents any of the topological descriptors mentioned above and *nB* is the numbers of bonds (without counting bond multiplicity) of the molecule.

For the case of the second PTML-MLP model (*Model 2*), the txt file containing the SMILES codes of the 9705 cases/chemicals was manually converted to *.smi, which was then transformed to *.sdf (no standardization options were applied) using the software Open Babel v2.4.0 [53]. Then, by using the software QuBiLs-MAS v1.0 (with the *.sdf file as the input) [54,55], we calculated the descriptors named atom-based local stochastic quadratic indices *ASq*m(*x*)*T* [54,56], where “m” was the *m*th power of the atom adjacency matrix and “*x*” was a physicochemical atomic property, such as *Hyd*, electronegativity (*E*), atomic weight (*Aw*), polarizability (*Pol*), *Psa*, and Kupchik’s vertex degree (*Ku*). The term “*T*” referred to the type of atom (aliphatic carbon, aromatic carbon, halogen, carbon in a methyl group, or heteroatoms, such as N, O, S, P, and Se) from which each *ASq*m(*x*)T was calculated.

To consider both the structure of any case/chemical and the experimental condition, *cj,* under which that case/chemical was tested, we applied a two-step approach, known as Box–Jenkins, which is the key aspect accounting for the great success of the PTML models [24,25,26,27,28,29,30,31,32,33,34,35,36,37,57,58,59,60,61,62]:

(2) $$avg [GTI]cj=\frac{1}{n\left(cj\right)}\times \overset{n\left(cj\right)}{\underset{i=1}{\sum }}GTI_{i}\;\;\;\;\;\;$$

In Equation (2), *GTI* refers to any of the molecular descriptors discussed above, i.e., *SM*(*PP*)k, *X*(*s*)o, *Xv*(*s*)o, *e*(*s*)o, *NTI*, and *ASq*m(*x*)T. The meanings of the terms *avg*(*GTI*)*cj* and *n*(*cj*) have already been explained in detail in a recent work [63]. This means that Equation (2) was applied to each element of the experimental conditions *cj* (that is, *ma*, *tg*, and *ei*) separately. Then, the second step of the Box–Jenkins approach was applied:

(3) $$D [GTI]cj=\left(\frac{GTI-avg [GTI]cj}{std\left(GTI\right)}\right)\cdot \sqrt{ps\left(cj\right)}\;\;\;\;\;\;$$

In Equation (3), *D*(*GTI*)*cj* is a descriptor that measures how much a chemical structurally and physicochemically deviates from a group of chemicals assayed by considering the same element of the experimental condition *cj*. On the other hand, *std*(*GTI*) is the standard deviation calculated from the *GTI* values; only chemicals in the training set were considered for the calculation of *std*(*GTI*). Last, *ps*(*cj*) represents the *a priori* probability of finding a case/chemical annotated as active by considering a defined element of *cj*. Thus, *ps*(*cj*) was calculated as follows:

(4) $$ps\left(cj\right)=\frac{n\left(cj\right)}{N_{T}\left(cj\right)}\;\;\;\;\;$$

In Equation (4), *n*(*cj*) has been defined in Equation (2) and represents the number of cases/chemicals assayed by considering the same element of the experimental condition *cj* [63], which were annotated as active (in the training set). Similarly, *N_T_*(*cj*), considering the same condition *cj*, represents the total number of cases/chemicals in the training set. We would like to emphasize that, as in the case of Equation (2), Equations (3) and (4) were applied to each element of the experimental condition *cj* (*ma*, *tg*, and *ei*) separately.

### 2.2. PTML Modeling, Applicability Domain, Descriptor Interpretation, Fragments, and Virtual Design

The creation and application of the two PTML-MLP models developed in this work involved steps such as splitting the dataset in the training and test series, selecting the most suitable *D*(*GTI*)*cj* descriptors using the software IMMAN v1.0 [64], analysis of the correlations among the *D*(*GTI*)*cj* descriptors via the Pearson correlation coefficient (*PCC*) [65], generation of the models using the program STATISTICA v13.5.0.17 [66], analysis of the applicability domain of each PTML-MLP model, interpretation of the *D*(*GTI*)*cj* descriptors, selection of suitable molecular fragments, and virtual design. All these steps have been described comprehensively in seminal works [37,63,67,68,69]. In any case, when selecting the best *D*(*GTI*)*cj* descriptors to subsequently build the PTML-MLP models, the mutual information differential Shannon’s entropy (MI-DSE) [70] and the Jeffreys information [71,72] were applied as criteria for descriptor selection; such criteria permitted the selection of at least one *D*(*GTI*)*cj* descriptor per each element of the experimental condition *cj*, which was a mandatory condition to develop the PTML-MLP models. When estimating the correlations via *PCC*, the interval −0.7 < *PCC* < 0.7 was used as a criterion of a lack of redundancy among the *D*(*GTI*)*cj* descriptors. We analyzed the global performance of the PTML-MLP models by relying on measures such as sensitivity (*Sn*(%)), specificity (*Sp*(%)), accuracy (*Ac*(%)), and the Matthews’ correlation coefficient (*MCC*) [73]. However, when choosing the most appropriate measure we determined the values of the local counterparts of (*Sn*(%)) and (*Sp*(%)), i.e., the local sensitivities (*Sn*(%))*ma*, (*Sn*(%))*tg*, and (*Sn*(%))*ei*, as well as the local specificities (*Sp*(%))*ma*, (*Sp*(%))*tg*, and (*Sp*(%))*ei*. Notice that these six local statistical indices depended on specific elements of the experimental condition *cj* (*ma*, *tg*, and *ei*) and we chose the PTML-MLP models displaying the highest values of the aforementioned local metrics.

## 3. Results and Discussion

### 3.1. PTML-MLP Models

We found that *Model 1* had the notation MLP 15-45-2, which means that this model was based on a multilayer perceptron network with 15 nodes in the input layer (number of *D*(*GTI*)*cj* descriptors present in the model), 45 neurons in the hidden layer, and two values of the categorical variable of inhibitory activity *IAi*(*cj*) were predicted in the output layer, i.e., active (*IAi*(*cj*) = 1) and inactive (*IAi*(*cj*) = –1). The same deduction can be made for *Model 2* whose notation was MLP 14-45-2. A summary of the *D*(*GTI*)*cj* descriptors present in each PTML-MLP model appears in Table 2, while information regarding the chemical and biological data used to build such models appears in Supplementary Materials S1 and S2, respectively.

Here, *Model 1* correctly classified 6312 out of the 7283 cases/chemicals in the training set (internal quality), which means *Ac*(%) = 86.67%. In the test set (predictive power), *Model 1* satisfactorily classified 2006 out of 2422 cases/chemicals, with *Ac*(%) = 82.82%. In the case of *Model 2*, similar results were achieved; this PTML-MLP model had *Ac*(%) values of 86.49% (6299 out of the 7283 cases/chemicals were correctly classified) and 81.75% (1980 out of 2422 cases/chemicals were correctly classified) for the training and test sets, respectively. Moreover, Table 3 shows that the two PTML-MLP models have high *Sp*(%) values, surpassing 80% in both the training and test set. In addition, for comparison, in Table 3 we have reported the values of the different statistical indices for the linear counterparts of our two PTML-MLP models. Such linear models were based on the technique known as linear discriminant analysis (LDA), and they respectively used the same variables (molecular descriptors of the type *D*(*GTI*)*cj*) from which our two PTML-MLP models were constructed.

From the analysis of the classification results in Table 3, it can be seen that each of our two PTML-MLP models outperforms its corresponding LDA counterpart. This suggests that the relationship between the measures of anti-PANC activity and the chemical structure of the molecules in the present dataset is modeled better with the use of non-linear machine learning algorithms, as in the case of our two PTML-MLP models.

Continuing with the results depicted in Table 3 for our PTML-MLP models, we can observe that for the case statistical index *Sn*(%), the values were higher than 80% in the training set while remaining above 75% in the test set. Moreover, in both PTML-MLP models, there is a strong convergence between the observed and the predicted values of the categorical variable of inhibitory activity *IAi*(*cj*) since the statistical metric *MCC* is closer to 1 than to 0 (random classifier) or –1 (completely erroneous prediction). Altogether, the results from Table 3 indicate that *Model 1* classified/predicted the current data slightly better than *Model 2*; however, *Model 2*, using one less *D*(*GTI*)*cj* descriptor than *Model 1* achieved similar performance.

We went deeper and, thus, analyzed the local metrics derived from *Sn*(%) and *Sp*(%). In this sense, *Model 1* exhibited values in the interval 69.64–99.21% for (*Sn*(%))*ma*, (*Sn*(%))*tg*, (*Sn*(%))*ei*, (*Sp*(%))*ma*, (*Sp*(%))*tg*, and (*Sp*(%))*ei* in the training set. The only exception was (*Sn*(%))*ei* = 33.33% for the assay information labeled as “F (assay format)”. In the test set, the behavior of *Model 1* was very similar to the training set, as the aforementioned local metrics were in the range of 64.29–100%. In this test set, *Model 1* maintained the same exception as in the training set and added the PANC cell line MIA-PaCa-2 with (*Sn*(%))*tg* = 55.56% and the assay information labeled as “B (assay format)” with (*Sp*(%))*ei* = 57.14%. In the case of *Model 2*, values were between 65.22% and 97.14% in the training set; a relatively low performance was associated with the PANC cell line PSN1, exhibiting (*Sn*(%))*tg* = 57.29% and the assay information annotated as “F (assay format)” displaying (*Sn*(%))*ei* = 44.44%. In the test set, *Model 2* achieved the interval 60.87–100% for the 6 local metrics mentioned above, with the exceptions being the labels of assay information “B (assay format)” with (*Sp*(%))*ei* = 53.97% and assay information labeled as “F (assay format)” with (*Sn*(%))*ei* = 38.46%. We would like to emphasize that most of the values reported in this work for (*Sn*(%))*ma*, (*Sn*(%))*tg*, (*Sn*(%))*ei*, (*Sp*(%))*ma*, (*Sp*(%))*tg*, and (*Sp*(%))*ei* were above 70%, which confirms the great capabilities of both PTML-MLP models to classify/predict complex biological data focused on anti-PANC activity by considering the 44 different experimental conditions, *cj* (as depicted in Table 1), employed in this study. All the details regarding the classification results of *Model 1* and *Model 2* can be found in Supplementary Materials S3 and S4, respectively.

Regarding the reliability of the predictions, we determined the AD of both PTML-MLP models according to the descriptor space approach (Supplementary Material S5), and, therefore, we calculated the total scores of the applicability domain (TSAD). For *Model 1*, only the cases/chemicals with TSAD = 15 were considered to be within the AD, with the number fifteen being equivalent to the number of *D*(*GTI*)*cj* descriptors present in *Model 1*. Only 2 of the 9705 cases/chemicals were found to be outside the AD of *Model 1* (TSAD < 15) and they both belonged to the test set. For *Model 2*, the ideal value TSAD = 14 indicated the ability of case/chemical to fall within the AD. In this model, only 8 of the 9705 cases/chemicals had TSAD < 14, thus remaining outside of the AD of *Model 2*.

Last, we would like to highlight the limitations and strengths of our two PTML-MLP models. A key limitation of our PTML-MLP models is their inability to correctly predict the entire dataset employed in this work. This means that the *D*(*GTI*)*cj* descriptors used to build the PTML-MLP models cannot characterize the whole complexity and molecular diversity in the present dataset. Another limitation is the one associated with the machine learning algorithm since none of the current algorithms are capable of encoding enough accurate chemical information. At least one of these two aspects is generalized to all computational models reported in the scientific literature. Consequently, in a virtual screening scenario, our PTML-MLP models will perform accurate predictions to some extent.

In any case, the strengths of our PTML-MLP models outweigh the aforementioned limitation. First, our PTML-MLP models are the first two models reported to date that can predict anti-PANC activity by simultaneously considering different mechanisms of action and multiple experimental conditions (involving dissimilar assay protocols) on the inhibition of both PANC-related proteins and PANC cell lines. This highlights the potentialities and wide applications of the PTML methodology in integrating chemical and biological data in the context of oncology research [24,25,26,74,75,76,77,78,79]. Second, our two PTML-MLP models are highly interpretable, physicochemically and structurally describing the data in terms of suitable molecular fragments (see Section 3.2), which can be useful to both computational and experimental chemists when selecting 2D pharmacophores. Third, by interpreting our PTML-MLP models, it is possible to design new anti-PANC molecules (see Section 3.3). Last, in contrast to most computational models, whose complex algorithms involve remarkable consumption of time and financial resources, our PTML-MLP models are cost-efficient; it only took two hours to create each of them when working on a portable computer (12 GB RAM).

### 3.2. Physicochemical and Structural Meanings of the Molecular Descriptors

Here, we are providing the physicochemical and structural interpretations of the *D*(*GTI*)*cj* descriptors in the PTML-MLP models. To support such interpretations, we employed certain graphics that illustrated the significance of each *D*(*GTI*)*cj* descriptor in the form of sensitivity values (SV) while extracting information on the propensities (increment or diminution) in the value of each *D*(*GTI*)*cj* descriptor [37,63,80]. Such propensities indicate how to vary the values of the *D*(*GTI*)*cj* descriptors to enhance the desired activity, which, in our case, is the improvement of the inhibitory activity against both the PANC-related proteins and the PANC cell lines.

#### 3.2.1. First PTML-MLP Model (Model 1)

**Figure 1:**
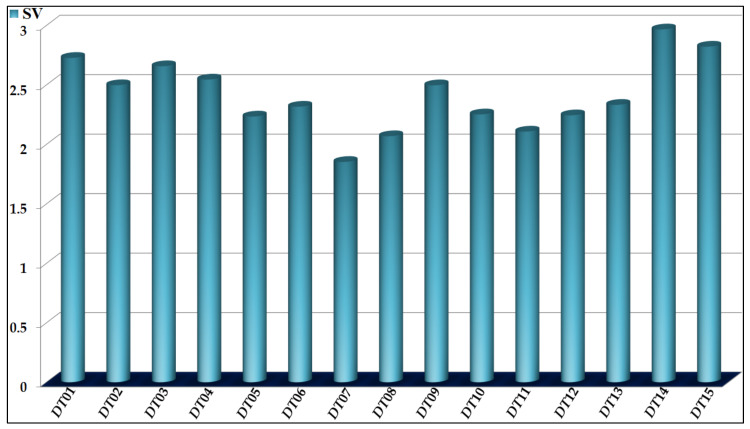
The different *D*(*GTI*)*cj* descriptors and their relative significances in *Model 1*.

The propensities of the *D*(*GTI*)*cj* descriptors in *Model 1* are reported in Table 4, while the relative importance of each of them (measured by *SV*) is depicted in Figure 1.

In *Model 1*, we have six *D*(*GTI*)*cj* descriptors derived from the so-called spectral moments of the bond adjacency matrix, which denote how much the physicochemical properties are distributed/concentrated throughout the chemical structure of a molecule [44,45,46,81,82,83]. These *D*(*GTI*)*cj* descriptors are *DTI*01, *DTI*06, *DTI*08, *DTI*09, *DTI*12, and *DTI*13 and among the most significant descriptors in *Model 1*, they rank third, ninth, fourteenth, seventh, eleventh, and eighth, respectively.

Three of these *D*(*GTI*)*cj* descriptors are derived from the spectral moments and are focused on hydrophobicity, according to the Ghose–Crippen approach [84]. Thus, *DTI*01 characterizes the decrease of the hydrophobicity in regions containing three-membered rings and fragments where one atom is attached to three other non-hydrogen atoms. Therefore, the presence of urea, carbamate, and carboxamide groups, as well as portions with secondary alcohols and amines, methylene moieties, and aziridine and oxirane rings favor a decrease in the value of *DTI*01. The same fragments mentioned in the case of *DTI*01, will also desirably decrease the value of *DTI*12; however, the latter of these *D*(*GTI*)*cj* descriptors will impact a molecule more globally. Another hydrophobicity-based descriptor is *DTI*06, which involves the increment of the hydrophobicity in regions in fragments containing seven bonds or less. The presence of three-membered rings and groups/moieties where an atom is bonded to four other non-hydrogen atoms (e.g., quaternary carbons, sulfonamides, and phosphorus-based functional groups, such as phosphine oxides, phosphonates, and phosphates) will increase the value of *DTI*06. At the same time, *DTI*08 describes the increase of the polar surface area, which can be achieved by increasing the number the functional groups (mainly based on nitrogen and oxygen atoms) capable of forming hydrogen bonds. On the other hand, *DTI*09 implies the augmentation of electronegative atoms in three-membered rings and groups where one atom is attached to two other non-hydrogen atoms. The functional groups described by *DTI*01 also favor *DTI*09, but the latter characterizes the structure in terms of the distribution of partial charges, also including the increase of halogens (particularly fluor). The last *D*(*GTI*)*cj* descriptor derived from the spectral moments is *DTI*13, which gives information regarding the diminution of the global polarizability in the molecules, thus favoring the presence of nitrogen, oxygen, and fluorine atoms, while diminishing the presence of aromatic rings or alkene moieties, as well as the functional groups attached to them.

In *Model 1*, we also have *D*(*GTI*)*cj* descriptors derived from atom-based connectivity indices, and, therefore, these *D*(*GTI*)*cj* descriptors measure the contribution of different fragments to the molecular accessibility [85,86], i.e., the ability of certain regions of a given molecule to interact with the surrounding chemical environment (e.g., with molecules such as water, amino acids in the cavity of a protein, and different components of the membrane PANC cell line). The *D*(*GTI*)*cj* descriptors are *DTI*02, *DTI*10, and *DTI*11, and, among the most influential, they ranked sixth, tenth, and thirteenth, respectively. Thus, in *Model 1*, *DTI*02 describes the increment of the molecular accessibility, mainly in linear (non-ramified) fragments containing at least one atom different from carbon, hydrogen, and boron. In contrast with *DTI*02, *DTI*10 measures the decrease of the average accessibility area of a molecule, which can be achieved by limiting the number of sulfur and halogen atoms (except fluor). In the case of *DTI*11, this descriptor indicates that augmenting the accessibility (and, therefore, the number) of six-membered rings will play a favorable role in enhancing the inhibitory activity against both the PANC-related proteins and the PANC cell lines.

Last, we have *D*(*GTI*)*cj* descriptors which encompass information content similar to that of the edge (bond) connectivity indices and, therefore, behave as measures of contribution to the molecular/molar volume [50,87,88]. Such *D*(*GTI*)*cj* descriptors under analysis are *DTI*03, *DTI*04, *DTI*05, *DTI*07, *DTI*14, and *DTI*15, and they have been considered as the fourth, fifth, twelfth, fifteenth, first, and second most significant descriptors in *Model 1*, respectively. Thus, *DTI*03 is a direct measure of the global volume of a molecule, expressing the decrease of this physicochemical property. This has important implications because diminishing the molecular volume means that a molecule will be able to better fit in the binding pocket of the PANC-related proteins while also allowing the molecule to be small enough to permeate the cellular membranes [89]. Decreasing the number of atoms in a molecule will favorably decrease the value of *DTI*03. A similar effect is expected to take place by diminishing the value of *DTI*04. The only difference is that *DTI*04 focuses on fragments formed by two bonds (without counting bond multiplicity). In the case of *DTI*05, this expresses the decrease of the volume in regions containing six-membered rings. We should highlight that this descriptor constrains *DTI*11, which was explained before. In the end, at least two six-membered rings can be part of the structure of a molecule, but these rings should have at least two substitutions while the presence of nitrogen atoms in these rings is a favorable factor. The descriptor *DTI*07 characterizes the increase of the molecular volume due to the presence of five-membered rings. The two most important *D*(*GTI*)*cj* descriptors (*DTI*14 and *DTI*15) indicate the increase of the average volume by decreasing the number of atoms in a molecule while increasing the number of regions without branching in that molecule. Particularly, *DTI*14 measures this effect in five-bond linear fragments while *DTI*15 focuses on six-bond fragments where the presence of a six-membered ring is also important.

#### 3.2.2. Second PTML-MLP Model (Model 2)

**Figure 2:**
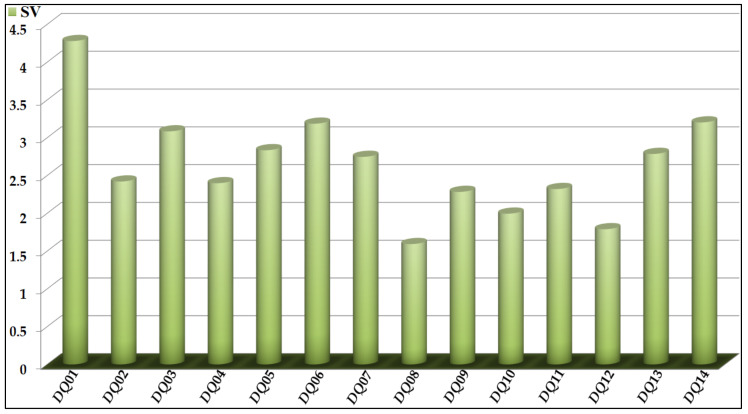
Relative importance of the different *D*(*GTI*)*cj* descriptors in *Model 2*.

As in the case of *Model 1*, data regarding each *D*(*GTI*)*cj* descriptor propensity and significance in *Model 2* can be found in Table 5 and Figure 2, respectively. The *D*(*GTI*)*cj* descriptors used in *Model 2* were derived from the atom-based local stochastic quadratic indices [54,56,90], which means that they characterize the distribution of different physicochemical properties of diverse fragments containing specific types of atoms [37,90]. In *Model 2*, seven of the fourteen *D*(*GTI*)*cj* descriptors are based on hydrophobicity, which indicates the paramount importance of this physicochemical property. These *D*(*GTI*)*cj* descriptors are *DQI*01, *DQI*02, *DQI*03, *DQI*09, *DQI*10, *DQI*12, and *DQI*14 and we would like to highlight that, these *D*(*GTI*)*cj* descriptors consider the multiplication of the hydrophobic contribution of a specific type of atom and the hydrophobic contribution of other non-hydrogen atoms situated at the given topological distance (number of bonds between any two atoms without counting bond multiplicity). Hence, instead of referring to an atomic hydrophobicity, we will use the term “joint hydrophobic contribution”.

In terms of significance in *Model 2*, the *D*(*GTI*)*cj* descriptors *DQI*01, *DQI*02, *DQI*03, *DQI*09, *DQI*10, *DQI*12, and *DQI*14 rank first, eighth, fourth, eleventh, twelfth, thirteenth, and second, respectively. By inspecting the descriptors *DQI*01, *DQI*09, and *DQI*10, we can see that they express the decrease of the joint hydrophobic contribution of any two atoms (with at least one of them being a halogen) separated by a topological distance of four, one, and two, respectively. Considering that all the halogens have a positive value of hydrophobic contribution [91], the atoms surrounding them must have a negative value of the hydrophobic contribution. Such atoms can be mainly primary and secondary carbons. Therefore, fragments such as 4-halocyclohexyl and 4-halobutyl will greatly and favorably diminish the values of *DQI*01, *DQI*09, and *DQI*10, thus enhancing the inhibitory activity against the PANC-related proteins and the PANC cell lines. At the same time, *DQI*02, *DQI*03, and *DQI*14 characterize an increase in the joint hydrophobic contribution of any two atoms, with one of them being a heteroatom (particularly N, O, S, or P). However, while *DQI*02 and *DQI*03 consider fragments where the heteroatom is at the topological distance of three and four (respectively) or less from any other non-hydrogen atom, *DQI*14 exclusively depends on the presence and number of heteroatoms. In any case, fragments containing the ethylenediamine or glycine moieties will desirably increase the value of *DQI*02, while fragments such as 1,3,5-triazin-2-amine, pyrimidin-2-amine, and heteroaliphatic rings (containing a nitrogen atom or being attached to it), as well as urea and carbamate, will do the same for *DQI*03; all these fragments will increase the value of *DQI*14. On the other hand, the descriptor *DQI*12 indicates the increase of the joint hydrophobic contribution between a carbon atom from a methyl group and its adjacent atoms (the topological distance equal to one). This means that to increase the value of *DQI*12, a carbon atom from a methyl group must be attached to a secondary (non-substituted) carbon or aminic nitrogen (resulting in a secondary amine).

In *Model 2*, we also have three *D*(*GTI*)*cj* descriptors containing electronic information. One of them is *DQI*04 (the ninth most influential), which characterizes the augmentation of the electronegativity of any two atoms (with one of them being a heteroatom) placed at the topological distance of two. Therefore, all the fragments mentioned for the descriptors *DQI*02 and *DQI*03 explained above cause a beneficial increase in the value of *DQI*04. In the case of *DQI*05 (the fifth most important), this describes the diminution of the polar surface area in any two adjacent heteroatoms. Because *DQI*05 directly depends on the presence of electron-rich heteroatoms, such as nitrogen and oxygen, to decrease the value of *DQI*05, two electron-rich heteroatoms should not be bonded. Moreover, in terms of electronic information, *DQI*08 expresses the increase of the polar surface area of any two heteroatoms placed at the topological distance of four or less. Therefore, all the fragments mentioned for the descriptor *DQI*03 (except for the heteroaliphatic rings) and in a lower degree, for *DQI*02, will increase the value of *DQI*08. Other groups containing sulfur (sulfonamides) and phosphorus (phosphates) will also increase the value of *DQI*08. This descriptor is the least significant in *Model 2*.

Steric factors play an essential role in enhancing the inhibitory activity of a chemical against the PANC-related proteins and the PANC cell lines. The *D*(*GTI*)*cj* descriptors accounting for the steric information are *DQI*06, *DQI*07, *DQI*11, and *DQI*13, and, regarding their influence in *Model 2*, they ranked third, seventh, tenth, and sixth, respectively. The descriptor *DQI*06 indicates the augmentation of the molecular size, mainly by increasing the number of aliphatic carbons but also by increasing the number of atoms (different from carbon) bonded to the aliphatic carbons. On the other hand, *DQI*07 means that the number of halogens must be decreased; if a halogen is present, iodine, bromine, and chlorine will be preferred over fluorine. In the case of *DQI*11, this characterizes the decrease of the atomic weight of any two atoms (with one of them being a halogen) placed at the topological distance of two. This means that to decrease the value of *DQI*11, the number of halogens should be reduced, and their chemical environments must be almost exclusively constituted by carbon atoms; halogens should be attached to aliphatic or aromatic rings or in the form of 2-haloethyl moieties. For *DQI*11, fluorine is strongly preferred over the other halogens. Last, we have *DQI*13, and its value can be increased by bonding carbons from methyl groups to low-polarizability atoms, such as nitrogen and oxygen. Therefore, the presence of methoxy and methylamino groups favor the increment of *DQI*13.

### 3.3. Designing Multi-Protein and Multi-Cell Inhibitors as Anti-PANC Agents

**Figure 3:**
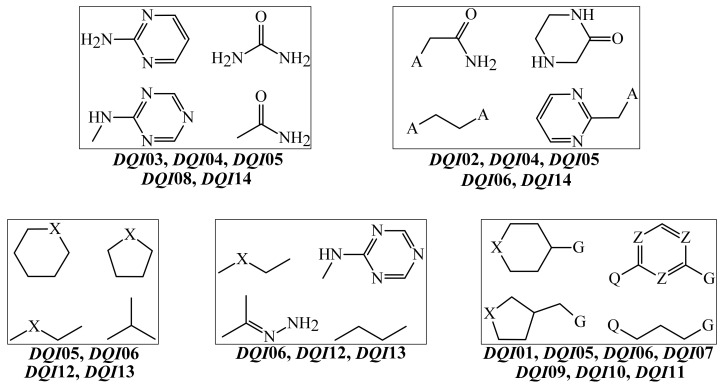
Generic molecular fragments directly extracted from the physicochemical and structural interpretation of the descriptors in *Model 2*. The descriptors are associated with different fragments. The symbols mean A = amino, hydroxyl, alkylamino, or alkoxy; G = halogen; Q = amino, hydroxyl, alkylamino, alkoxy, or a non-substituted secondary carbon; X = O, -NH-, or a secondary carbon; Z = N or aromatic carbon.

The guidelines applied to the design of new molecules from PTML models have been reported before [25,26,28,67,80,92]. Here, we used *Model 2* as a tool to design new molecules from the fragments present in Figure 3.

There were two reasons for which we preferred *Model 2* over *Model 1*. From one side, as mentioned in Section 3.1, *Model 2*, with one less descriptor, achieved a statistical performance comparable to that of *Model 1*. On the other hand, from a physicochemical point of view, *Model 2* offers a simpler (but more detailed) explanation than *Model 1*. The joint interpretation of the *D*(*GTI*)*cj* descriptors in *Model 2* suggests the presence of three well-defined regions that seem to be essential when designing a molecule. First, there should be a hydrophilic region containing several heteroatoms that can interact through hydrogen bonds. The second region, situated in the center of the molecule, may also contain heteroatoms but these should be more dispersed, with any two heteroatoms separated at a distance of three bonds. In this region, aliphatic atoms may serve as “bridges” between any two heteroatoms. The third region is expected to be hydrophobic, particularly containing a 4-halophenyl or 4-halocyclohexyl moiety, which means that the halogen will be in the periphery of the molecule.

**Figure 4:**
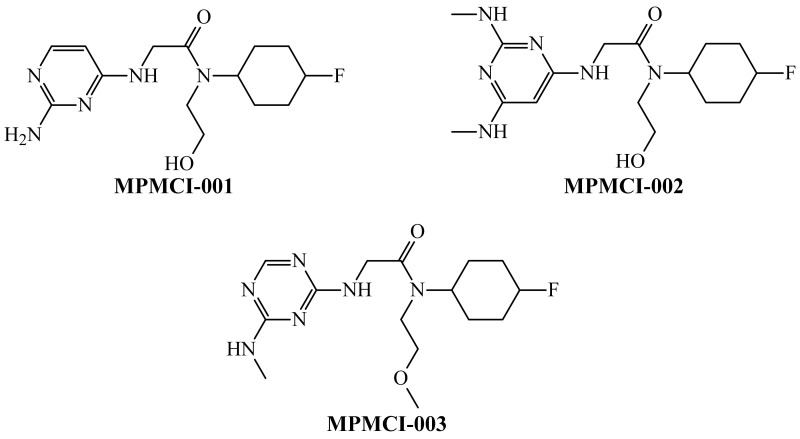
New molecules designed from suitable molecular fragments by using the physicochemical and structural interpretations as guidelines.

By combining the aforementioned joint interpretation with the inspection of the fragments in Figure 3, we designed three structurally related molecules, which are depicted in Figure 4.

The three designed molecules were predicted by *Model 1* and *Model 2* to be anti-PANC agents through the inhibition of the PANC-related proteins and PANC cell lines. However, there were differences in the predicted probabilities. For instance, for all the 44 experimental conditions *cj* reported in this work, MPMCI-001 and MPMCI-003 were predicted by both PTML-MLP models with probability values close to 100%. However, in the case of MPMCI-002, this happened only with *Model 2*. That does not mean that *Model 1* poorly predicted MPMCI-002. Notice that *Model 1* predicted MPMCI-002 to be an anti-PANC agent in 42 of the 44 experimental conditions, but the predicted probability values were lower when compared to those obtained for MPMCI-001 and MPMCI-003. This means that, in the structure of MPMCI-002, the methyl group from the methylamino moiety located between the two nitrogen atoms of the pyrimidine ring should be removed. The difference in the predicted probabilities also demonstrates the sensibility of the *D*(*GTI*)*cj* descriptors in *Model 1*, allowing this model to be used as a filter of *Model 2* to ensure the correct design of new molecules. In any case, the fact that the three designed molecules were predicted by both PTML-MLP models as multi-protein and multi-cell inhibitors, theoretically confirms the aforementioned molecules to be anti-PANC agents. Moreover, the three molecules were within the applicability domain of these models. All the results of the predictions (including the assessment of the applicability domain) performed by *Model 1* and *Model 2* for the design molecules can be found in Supplementary Materials S6 and S7, respectively.

We searched for our designed molecules in different databases, such as ChEMBL [41,93], ZINC [94], and eMolecules [95], to check their novelty. In this sense, we used the similarity cutoff of 80%. No molecules similar to those designed by us were found. We would like to add that we also calculated several global physicochemical properties for the designed molecules (Table 6), which allowed us to have an idea regarding their drug-likeness.

In doing so, the calculated physicochemical properties allowed the use of three approaches known as the Lipinski rule of five [96], Ghose filter [97], and Veber’s guidelines [98]. We compared the values of each physicochemical property for each of the designed molecules with the cutoff values/intervals established by each of the aforementioned filters. Our designed molecules complied with all these criteria.

## 4. Conclusions

In the context of therapeutic solutions for oncology research, finding anti-PANC chemicals is one of the greatest challenges. The multi-factorial nature of PANC is a clear indicator that both experimental and computational approaches must be focused on searching for multi-target agents to more efficiently limit or eradicate PANC growth through multiple mechanisms of action. Our computational methodology, relying on two PTML-MLP models, has established the theoretical basis to enable the fragment-based design of new molecular entities that can act as multi-protein and multi-cell inhibitors against PANC. This work consolidates the usefulness of PTML modeling as a strategy that can yield potentially new anticancer therapeutics exhibiting adequate drug-like properties.

## Figures and Tables

**Table 1:** Experimental conditions reported in this work.

*ma* ^a^	Cutoff ^b^	*tg* ^c^	*ei* ^d^
IC_50_ (nM)p	≤1100 nM	Caspase-1	B (assay format)
		Caspase-1	B (single protein format)
		Caspase-1	B (cell-based format)
	≤1635 nM	TNF-alpha	B (single protein format)
		TNF-alpha	F (assay format)
		TNF-alpha	B (assay format)
		TNF-alpha	B (cell-based format)
		TNF-alpha	F (cell-based format)
	≤50 nM	IGF1R	B (single protein format)
		IGF1R	B (cell-based format)
		IGF1R	B (assay format)
		IGF1R	F (cell-based format)
		IGF1R	F (assay format)
IC_50_ (nM)c	≤6449.735 nM	PSN1	F (cell-based format)
		PANC-03-27	F (cell-based format)
		HPAC	F (cell-based format)
		MZ1-PC	F (cell-based format)
		KP-4	F (cell-based format)
		KP-2	F (cell-based format)
		PA-TU-8988T	F (cell-based format)
		Capan-2	F (cell-based format)
		MIA-PaCa-2	F (cell-based format)
		CFPAC-1	F (cell-based format)
		PANC-10-05	F (cell-based format)
		BxPC-3	F (cell-based format)
		SUIT-2	F (cell-based format)
		KP-1N	F (cell-based format)
		HuP-T4	F (cell-based format)
		SW1990	F (cell-based format)
		PL18	F (cell-based format)
		QGP-1	F (cell-based format)
		HuP-T3	F (cell-based format)
		SU8686	F (cell-based format)
		PL4	F (cell-based format)
		PA-TU-8902	F (cell-based format)
		PANC-02-03	F (cell-based format)
		DAN-G	F (cell-based format)
		CAPAN-1	F (cell-based format)
		PANC-08-13	F (cell-based format)
		HPAF-II	F (cell-based format)
		KP-3	F (cell-based format)
		YAPC	F (cell-based format)
		AsPC-1	F (cell-based format)
		PANC-04-03	F (cell-based format)

^a^ Measure of biological activity; IC_50_ (nM)p is the concentration required for 50% inhibition of a protein, while IC_50_ (nM)c is the concentration required for a chemical to inhibit cell viability by 50%. ^b^ Value of activity from which a molecule was labeled and considered as active (*IAi*(*cj*) = 1). ^c^ Refers to the targets (either a protein or a PANC cell line). ^d^ Information related to the diverse experimental assays. Here, each annotation is a combination of the columns “assay type” (first letter) and “BioAssay Ontology” (phrase between parentheses), which are reported in any ChEMBL file containing bioactivity data. Each assay involving a PANC cell line was annotated as “F (cell-based format)”.

**Table 2:** Molecular descriptors of the type *D*(*GTI*)*cj* present in the PTML-MLP models.

Model ^a^	Symbology ^b^	Code ^c^	Concept
*Model 1*	*D*(*NSM*(*Hyd*)3)*ma*	*DT*01	Deviation of the normalized spectral moment of order 3 based on hydrophobicity-weighted bonds.
	*D*(*NXv*(*P*)4)*ma*	*DT*02	Deviation of the normalized Kier–Hall (valence) connectivity index involving only path-based subgraphs of order 4.
	*D*(*Ne*(*P*)1)*ma*	*DT*03	Deviation of the normalized edge (bond) connectivity index involving only path-based subgraphs of order 1.
	*D*(*Ne*(*P*)2)*ma*	*DT*04	Deviation of the normalized edge (bond) connectivity index involving only path-based subgraphs of order 2.
	*D*(*Ne*(*Ch*)6)*ma*	*DT*05	Deviation of the normalized edge (bond) connectivity index involving only chain-based subgraphs of order 6.
	*D*(*SM*(*Hyd*)7)*tg*	*DT*06	Deviation of the spectral moment of order 7 based on hydrophobicity-weighted bonds.
	*D*(*e*(*Ch*)5)*tg*	*DT*07	Deviation of the edge (bond) connectivity index involving only chain-based subgraphs of order 5.
	*D*(*NSM*(*Psa*)1)*tg*	*DT*08	Deviation of the normalized spectral moment of order 1 based on bonds weighted by the polar surface area.
	*D*(*NSM*(*Gas*)3)*tg*	*DT*09	Deviation of the normalized spectral moment of order 3 based on bonds weighted by the Gasteiger–Marsili charges.
	*D*(*NXv*(*P*)1)*tg*	*DT*10	Deviation of the normalized Kier-Hall (valence) connectivity index involving only path-based subgraphs of order 1.
	*D*(*Xv*(*Ch*)6)*ei*	*DT*11	Deviation of the Kier-Hall (valence) connectivity index involving only chain-based subgraphs of order 6.
	*D*(*NSM*(*Hyd*)1)*ei*	*DT*12	Deviation of the normalized spectral moment of order 1 based on hydrophobicity-weighted bonds.
	*D*(*NSM*(*Mol*)1)*ei*	*DT*13	Deviation of the normalized spectral moment of order 1 based on bonds weighted by the molar refractivity.
	*D*(*Ne*(*P*)5)*ei*	*DT*14	Deviation of the normalized edge (bond) connectivity index involving only path-based subgraphs of order 5.
	*D*(*Ne*(*PC*)6)*ei*	*DT*15	Deviation of the normalized edge (bond) connectivity index involving only path-cluster subgraphs of order 6.
*Model 2*	*D*(*ASq*4(*Hyd*)*G*)*ma*	*DQ*01	Deviation of the stochastic atom-based local quadratic index weighted by the hydrophobicity of the halogens and their neighbor atoms located at the topological distance of 4.
	*D*(*ASq*3(*Hyd*)*Y*)*ma*	*DQ*02	Deviation of the stochastic atom-based local quadratic index weighted by the hydrophobicity of the heteroatoms (N, O, S, P, and Se) and their neighbor atoms located at the topological distance of 3.
	*D*(*ASq*4(*Hyd*)*Y*)*ma*	*DQ*03	Deviation of the stochastic atom-based local quadratic index weighted by the hydrophobicity of the heteroatoms (N, O, S, P, and Se) and their neighbor atoms located at the topological distance of 4.
	*D*(*ASq*2(*E*)*Y*)*ma*	*DQ*04	Deviation of the stochastic atom-based local quadratic index weighted by the electronegativity of the heteroatoms (N, O, S, P, and Se) and their neighbor atoms located at the topological distance of 2.
	*D*(*ASq*1(*Psa*)*Y*)*ma*	*DQ*05	Deviation of the stochastic atom-based local quadratic index weighted by the polar surface area of the heteroatoms (N, O, S, P, and Se) and their neighbor atoms located at the topological distance of 1.
	*D*(*ASq*1(*Aw*)*C*)*tg*	*DQ*06	Deviation of the stochastic atom-based local quadratic index weighted by the atomic weight of the aliphatic carbons and their neighbor atoms located at the topological distance of 1.
	*D*(*ASq*0(*Ku*)*G*)*tg*	*DQ*07	Deviation of the stochastic atom-based local quadratic index (order 0) weighted by the Kupchik’s vertex degree of the halogens in a molecule.
	*D*(*ASq*4(*Psa)Y*)*tg*	*DQ*08	Deviation of the stochastic atom-based local quadratic index weighted by the polar surface area of the heteroatoms (N, O, S, P, and Se) and their neighbor atoms located at the topological distance of 4.
	*D*(*ASq*1(*Hyd*)*G*)*ei*	*DQ*09	Deviation of the stochastic atom-based local quadratic index weighted by the hydrophobicity of the halogens and their neighbor atoms located at the topological distance of 1.
	*D*(*ASq*2(*Hyd*)*G*)*ei*	*DQ*10	Deviation of the stochastic atom-based local quadratic index weighted by the hydrophobicity of the halogens and their neighbor atoms located at the topological distance of 2.
	*D*(*ASq*2(*Aw*)*G*)*ei*	*DQ*11	Deviation of the stochastic atom-based local quadratic index weighted by the atomic weight of the halogens and their neighbor atoms located at the topological distance of 2.
	*D*(*ASq*1(*Hyd*)*M*)*ei*	*DQ*12	Deviation of the stochastic atom-based local quadratic index weighted by the hydrophobicity of the aliphatic carbons (only methyl groups) and their neighbor atoms located at the topological distance of 1.
	*D*(*ASq*1(*Ku*)*M*)*ei*	*DQ*13	Deviation of the stochastic atom-based local quadratic index weighted by the Kupchik’s vertex degree of the aliphatic carbons (only methyl groups) and their neighbor atoms located at the topological distance of 1.
	*D(ASq*0(*Hyd*)*Y*)*ei*	*DQ*14	Deviation of the stochastic atom-based local quadratic index (order 0) weighted by the hydrophobicity heteroatoms (N, O, S, P, and Se) in a molecule.

^a^*Model 1*, first PTML-MLP model, which contains the first 15 *D*(*GTI*)*cj* descriptors shown in this table; *Model 2*, second PTML-MLP model, which contains the remaining14 *D*(*GTI*)*cj* descriptors shown in this table. ^b^ Molecular descriptors of the type *D*(*GTI*)*cj* with endings on “*ma*” consider both the molecular structure and the measure of inhibitory activity. Those with the ending “*tg*” depend on the molecular structure and the biological target (either a protein or a PANC cell line). Finally, *D*(*GTI*)*cj* descriptors with the ending “*ei*” characterize the molecular structure and information on the diverse experimental assays. ^c^ Codes were used to abbreviate the representation of the *D*(*GTI*)*cj* descriptors.

**Table 3:** Statistical indices demonstrating the performances of the two PTML-MLP models.

SYMBOLS ^a,b^	*Model 1*		*Model 2*	
	Training Set	Test Set	Training Set	Test Set
*N*_Active_	3010	1001	3010	1001
*CCC*_Active_	2495 (1293)	799 (447)	2486 (1084)	785 (352)
*Sn*(%)	82.89% (42.96%)	79.82% (44.66%)	82.59% (36.01%)	78.42% (35.16%)
*N*_Inactive_	4273	1421	4273	1421
*CCC*_Inactive_	3817 (3693)	1207 (1219)	3813 (3625)	1195 (1194)
*Sp*(%)	89.33% (86.43%)	84.94% (85.78%)	89.23% (84.84%)	84.10% (84.03%)
*MCC*	0.724 (0.331)	0.646 (0.338)	0.721 (0.241)	0.624 (0.222)

^a^*N*_Active_, Number of chemicals/cases annotated as active; *N*_Inactive_, Number of chemicals/cases designated as inactive; *CCC*_Active_, Number of chemicals/cases correctly classified/predicted as active; *CCC*_Inactive_, Number of chemicals/cases correctly classified/predicted as inactive; *Sn*(%), Sensitivity (percentage of chemicals/cases correctly classified as active); *Sp*(%), Specificity (percentage of chemicals/cases properly classified as inactive); *MCC*, Refers to the Matthews’ correlation coefficient. ^b^ Values between parentheses correspond to models derived from the technique known as linear discriminant analysis (LDA).

**Table 4:** Molecular descriptors of the type *D*(*GTI*)*cj* present in the first PTML-MLP model (*Model 1*) and their relative propensities.

Codes ^a^	Descriptors	CLASS-BASED MEANS ^b^		Propensity ^c^
		Active	Inactive	
*DTI*01	*D*(*NSM*(*Hyd*)3)*ma*	−2.3485 × 10^−2^	1.0631 × 10^−1^	Decrease
*DTI*02	*D*(*NXv*(*P*)4)*ma*	6.6912 × 10^−3^	5.5922 × 10^−3^	Increase
*DTI*03	*D*(*Ne*(*P*)1)*ma*	−3.2309 × 10^−4^	8.6125 × 10^−2^	Decrease
*DTI*04	*D*(*Ne*(*P*)2)*ma*	−4.7514 × 10^−2^	1.4992 × 10^−1^	Decrease
*DTI*05	*D*(*Ne*(*Ch*)6)*ma*	−4.1335 × 10^−2^	1.7654 × 10^−1^	Decrease
*DTI*06	*D*(*SM*(*Hyd*)7)*tg*	3.1706 × 10^−2^	−2.5668 × 10^−1^	Increase
*DTI*07	*D*(*e*(*Ch*)5)*tg*	5.2858 × 10^−3^	−4.9427 × 10^−2^	Increase
*DTI*08	*D*(*NSM*(*Psa*)1)*tg*	2.2379 × 10^−2^	−1.9481 × 10^−2^	Increase
*DTI*09	*D*(*NSM*(*Gas*)3)*tg*	2.8122 × 10^−2^	−1.3897 × 10^−1^	Increase
*DTI*10	*D*(*NXv*(*P*)1)*tg*	−1.2919 × 10^−2^	1.1367 × 10^−1^	Decrease
*DTI*11	*D*(*Xv*(*Ch*)6)*ei*	−4.9125 × 10^−3^	−7.1198 × 10^−2^	Increase
*DTI*12	*D*(*NSM*(*Hyd*)1)*ei*	−5.5366 × 10^−2^	2.3959 × 10^−1^	Decrease
*DTI*13	*D*(*NSM*(*Mol*)1)*ei*	−4.2905 × 10^−2^	2.2042 × 10^−1^	Decrease
*DTI*14	*D*(*Ne*(*P*)5)*ei*	−1.2401 × 10^−2^	−5.6792 × 10^−2^	Increase
*DTI*15	*D*(*Ne*(*PC*)6)*ei*	3.4465 × 10^−2^	−2.3273 × 10^−1^	Increase

^a^ Symbols of the different molecular descriptors of the type *D*(*GTI*)*cj* in *Model 1* as represented in Table 2. ^b^ Average values of each *D*(*GTI*)*cj* descriptor by considering the active and inactive categories. ^c^ Relative tendency of a molecular descriptor to vary (increase or decrease) its value, resulting in a simultaneous enhancement of the inhibitory activity against PANC-related proteins (caspase-1, TNF-alpha, and IGF1R) and the PANC cell lines.

**Table 5:** Class-based means and relative propensities of the *D*(*GTI*)*cj* descriptors present in the second PTML-MLP model (*Model 2*).

Codes ^a^	Descriptors	CLASS-BASED MEANS ^b^		Propensity ^c^
		Active	Inactive	
*DQI*01	*D*(*ASq*4(*Hyd*)*G*)*ma*	−5.4750 × 10^−3^	−4.6447 × 10^−3^	Decrease
*DQI*02	*D*(*ASq*3(*Hyd*)*Y*)*ma*	2.6348 × 10^−2^	−1.8596 × 10^−1^	Increase
*DQI*03	*D*(*ASq*4(*Hyd*)*Y*)*ma*	3.5673 × 10^−2^	−1.3724 × 10^−1^	Increase
*DQI*04	*D*(*ASq*2(*E*)*Y*)*ma*	5.0259 × 10^−2^	−2.2849 × 10^−1^	Increase
*DQI*05	*D*(*ASq*1(*Psa*)*Y*)*ma*	3.8557 × 10^−3^	6.3789 × 10^−2^	Decrease
*DQI*06	*D*(*ASq*1(*Aw*)*C*)*tg*	5.9744 × 10^−2^	−3.4756 × 10^−1^	Increase
*DQI*07	*D*(*ASq*0(*Ku*)*G*)*tg*	3.9557 × 10^−3^	1.1249 × 10^−1^	Decrease
*DQI*08	*D*(*ASq*4(*Psa)Y*)*tg*	3.1247 × 10^−2^	−1.2646 × 10^−1^	Increase
*DQI*09	*D*(*ASq*1(*Hyd*)*G*)*ei*	−1.1160 × 10^−2^	4.0920 × 10^−2^	Decrease
*DQI*10	*D*(*ASq*2(*Hyd*)*G*)*ei*	5.8725 × 10^−4^	3.3683 × 10^−2^	Decrease
*DQI*11	*D*(*ASq*2(*Aw*)*G*)*ei*	9.2750 × 10^−3^	1.9038 × 10^−2^	Decrease
*DQI*12	*D*(*ASq*1(*Hyd*)*M*)*ei*	2.6208 × 10^−2^	−1.7829 × 10^−1^	Increase
*DQI*13	*D*(*ASq*1(*Ku*)*M*)*ei*	2.8400 × 10^−2^	−3.0151 × 10^−1^	Increase
*DQI*14	*D(ASq*0(*Hyd*)*Y*)*ei*	1.3478 × 10^−2^	−2.1995 × 10^−2^	Increase

^a^ Symbols of the different *D*(*GTI*)*cj* descriptors in the second PTML-MLP model according to Figure 2. ^b^ Average values of each *D*(*GTI*)*cj* descriptor by considering the active and inactive categories. ^c^ Relative tendency of each *D*(*GTI*)*cj* descriptor to vary (increase or decrease) its value, resulting in a simultaneous enhancement of the inhibitory activity against PANC-related proteins (caspase-1, TNF-alpha, and IGF1R) and the PANC cell lines.

**Table 6:** Physicochemical properties estimated for the designed molecules.

ID ^a^	nHDon	nHAcc	MW (Da)	MlogP	AlogP	MR (cm^3^/mol)	nAT	RBN	PSA (Å)
MPMCI-001	4	8	311.41	1.429	0.675	83.009	44	6	104.37
MPMCI-002	4	9	354.49	2.259	1.578	99.009	52	8	102.41
MPMCI-003	2	9	340.46	2.151	1.515	92.717	49	8	92.27

^a^ The physicochemical properties described in this table are as follows: number of hydrogen bond donors (nHDon), number of hydrogen bond acceptors (nHAcc), molecular weight (MW), logarithm of the octanol/water partition coefficient according to the Moriguchi approach (MlogP), logarithm of the octanol/water partition coefficient according to the Ghose–Crippen approach (AlogP), molar refractivity according to the Ghose–Crippen approach (MR), total number of atoms (nAT), number of rotatable bonds (RBN), and polar surface area (PSA).

## Data Availability

All the chemical and biological (raw) data were retrieved from the public repository known as ChEMBL (https://www.ebi.ac.uk/chembl/, accessed on 4 January 2022).

## Supplementary Materials

The following are available online at https://www.mdpi.com/article/10.3390/biomedicines10020491/s1, Supplementary Material S1: Topological indices, averages, and standard deviation values for *Model 1*, Supplementary Material S2: Atom-based local stochastic quadratic indices, averages, and standard deviation values for *Model 2*, Supplementary Material S3: *D*(*GTI*)*cj* descriptors, classification results, and local metrics for *Model 1*, Supplementary Material S4: *D*(*GTI*)*cj* descriptors, classification results, and local metrics for *Model 2*, Supplementary Material S5: Applicability domain of the two PTML-MLP models by considering the cases/chemicals belonging to both the training and test sets, Supplementary Material S6: Topological indices, *D*(*GTI*)*cj* descriptors, classification results, and applicability domain of the designed molecules according to *Model 1*, Supplementary Material S7: Atom-based local stochastic quadratic indices, *D*(*GTI*)*cj* descriptors, classification results, and applicability domain of the designed molecules according to *Model 2*.
